# Using Application Programming Interfaces (APIs) to Access Google Data and Gain Insights Into Searches on Birth Control in Louisiana and Mississippi, 2014-2018: Infoveillance Study

**DOI:** 10.2196/25923

**Published:** 2021-07-12

**Authors:** Ndola Prata, Karen Weidert, Anne Zepecki, Elina Yon, Elizabeth Pleasants, Petrice Sams-Abiodun, Sylvia Guendelman

**Affiliations:** 1 Bixby Center for Population, Health and Sustainability School of Public Health University of California, Berkeley Berkeley, CA United States; 2 The Wallace Center for Maternal, Child, and Adolescent Health School of Public Health University of California, Berkeley Berkeley, CA United States; 3 College of Engineering University of California, Berkeley Berkeley, CA United States; 4 Planned Parenthood Gulf Coast New Orleans, LA United States

**Keywords:** birth control, search data, Google Trends, infoveillance, infodemiology, Louisiana, Mississippi

## Abstract

**Background:**

It is now common to search for health information online. A 2013 Pew Research Center survey found that 77% of online health seekers began their query at a search engine. The widespread use of online health information seeking also applies to women’s reproductive health. Despite online interest in birth control, not much is known about related interests and concerns reflected in the search terms in the United States.

**Objective:**

In this study, we identify the top search terms on Google related to birth control in Louisiana and Mississippi and compare those results to the broader United States, examining how Google searches on birth control have evolved over time and identifying regional variation within states.

**Methods:**

We accessed search data on birth control from 2014-2018 from 2 Google application programming interfaces (APIs), Google Trends and Google Health Trends. We selected Google as it is the most commonly used search engine. We focused our analysis on data from 2017 and compared with 2018 data as appropriate. To assess trends, we analyzed data from 2014 through 2018. To compare the relative search frequencies of the top queries across Louisiana, Mississippi, and the United States, we used the Google Health Trends API. Relative search volume by designated marketing area (DMA) gave us the rankings of search volume for each birth control method in each DMA as compared to one another.

**Results:**

Results showed that when people searched for “birth control” in Louisiana and the broader United States, they were searching for information on a diverse spectrum of methods. This differs from Mississippi, where the data indicated people were mainly searching for information related to birth control pills. Across all locations, searches for birth control pills were significantly higher than any other queries related to birth control in the United States, Louisiana, and Mississippi, and this trend remained constant from 2014 to 2018. Regional level analysis showed variations in search traffic for birth control across each state.

**Conclusions:**

The internet is a growing source of health information for many users, including information on birth control. Understanding popular Google search queries on birth control can inform in-person discussions initiated by family planning practitioners and broader birth control messaging campaigns.

**International Registered Report Identifier (IRRID):**

RR2-10.2196/16543

## Introduction

The use of the internet to source health information has become increasingly popular. It is now common to seek information online prior to, or in some cases, instead of meeting with health professionals [1-3]. A 2013 Pew Research Center survey found that 77% of online health seekers began their query at a search engine [4]. The widespread use of online health information seeking also applies to women’s reproductive health [5,6]. One study estimated women’s most prevalent health concerns by examining commonly searched health-related keywords in the Google search engine between 2012 and 2013 in the United States. The authors found contraception among the top 25 monthly searched keywords of all categories, with 27,100 related searches estimated each month [5]. These searches were more common in states with the highest number of abortion restrictions. Similarly, a study found that between 2011 and 2015, the number of Google searches in the United States using terms related to self-abortion increased from 119,000 to 700,000 [7], a period of time when states enacted many anti-abortion measures [8]. A qualitative study on the role of social networks in contraceptive decision making revealed that two-thirds of participants used the internet to find information on contraception, noting the privacy of the internet made it an ideal resource [9].

Despite online interest in birth control, not much is known about related interests and concerns reflected in the search terms of the United States population. Online searches represent a novel source of data for understanding contraception needs among internet users. Additionally, the needs for online information and contraceptive resources may be changing as reproductive health policies increasingly restrict access and funding for birth control services and information [7,8,10,11]. In recent years, legislative and regulatory attacks against sexual and reproductive health have reduced the availability of family planning providers. Title X, the only United States federal program dedicated to providing family planning services for low-income people, has narrowed the network of providers by excluding those that offer abortion [7,10].

This study explores online Google search traffic related to birth control in 2 states: Louisiana and Mississippi. We selected these states for our study because they have some of the poorest reproductive health indicators in the country as well as large rural populations and communities of color that worsen their barriers to in-person contraceptive care [12,13]. These 2 states also count on limited data to document population-level contraceptive preferences and concerns [14-16]. Through application of our protocol, we identified the search topics related to birth control that are most often searched on Google in Louisiana and Mississippi and compared those results to the United States as a whole, examining how the searches have changed over time. Furthermore, we examined variations in birth control searches across geographic areas within each state to explore how search interest for each contraceptive method varies between different geographical areas. We hypothesized that understanding the information people are seeking online can help strengthen the capacity of key organizations and reproductive health leaders to deliver appropriate, acceptable, client-centered, and high-quality contraceptive services. Data on the contraceptive methods most searched by geolocation can inform information, education, and communications campaigns and ensure potential users are getting clear information on various methods.

## Methods

We accessed search data on birth control from 2014 to 2018 from 2 Google application programming interfaces (APIs), Google Trends and Google Health Trends. We selected Google as it is the most commonly used search engine [17]. We focused our analysis on data from 2017, and comparisons with 2018 data were made as appropriate. To assess trends, we analyzed data from 2014 through 2018.

**Figure 1:**
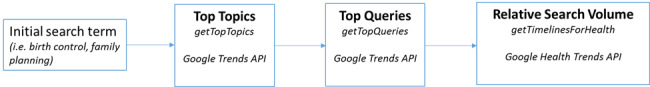
Steps in retrieving data from custom Google application programming interfaces (APIs).

Figure 1 illustrates the steps in retrieving data from the Google APIs. We followed a methodological framework to retrieve Google data from multiple APIs [18]. Google Trends API allowed us to retrieve search queries linked to the initial search term “birth control” for a given location in 2017.

**Figure 2:**
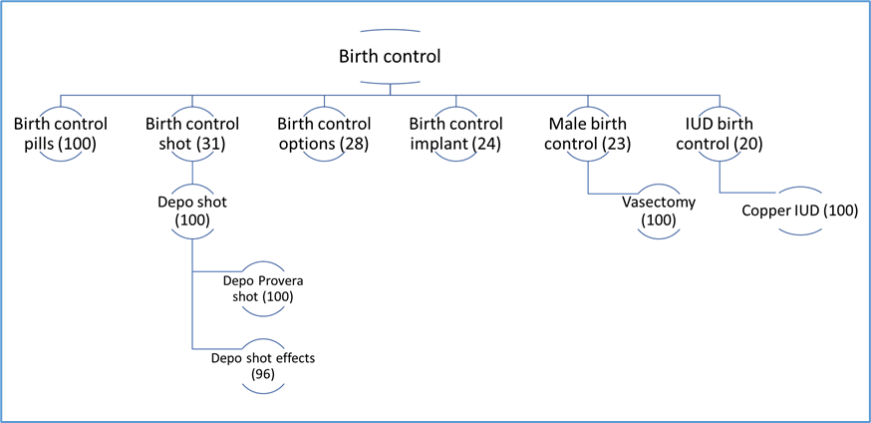
Directed graph of the top birth control–related search queries in the United States in 2017, with the number value representing the association between the search query and the top topic (birth control), with 100 reflecting a high association.

**Figure 3:**
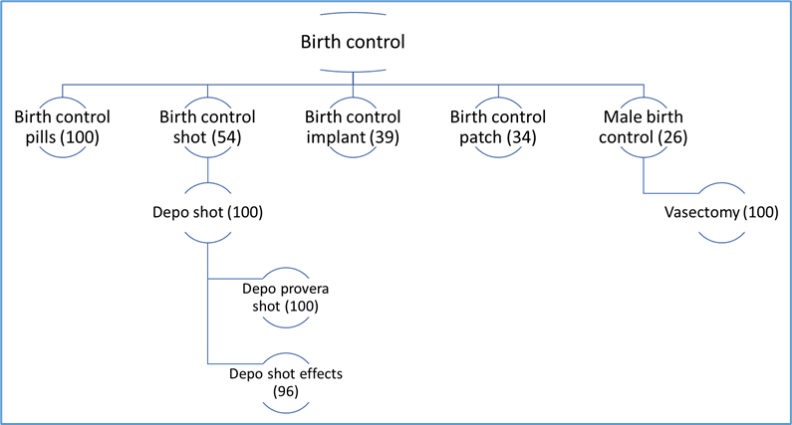
Directed graph of the top birth control–related search queries in Louisiana in 2017, with the number value representing the association between the search query and the top topic (birth control), with 100 reflecting a high association.

**Figure 4:**
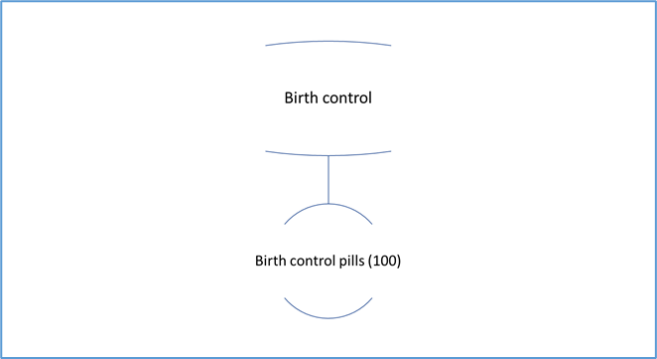
Directive graph of the top birth control–related search queries in in Mississippi in 2017, with the number value representing the association between the search query and the top topic (birth control), with 100 reflecting a high association.

We arrived at “birth control” as our preferred search term after implementing a simulation methodology [18] that showed very strong association with birth control methods, compared to weak or no association between “contraception” or “family planning” with birth control methods. The Google Trends API returns the top queries a user searches along with a relative search index ranging from 0 to 100 (with 100 denoting the query with the strongest association to the initial search term and 0 denoting the weakest association). The Google Health Trends API normalizes search data to make comparisons between queries easier. Search results are normalized to the time and location of a query, by dividing each data point by the total searches of the geography and time range it represents to compare relative volume. The resulting numbers are then scaled on a range of 0 to 100 based on a topic’s proportion to all searches on all topics. Google Trends does filter out some types of searches, such as duplicate searches done by the same person and those made by very few people; Google Trends only shows data for popular terms, so search terms with low volume appear as “0” [19]. Top queries are not displayed when the search volume for a given query falls below what Google considers the threshold of traffic volume (search terms are assigned a value of 0); the search volume often does not meet this threshold in less densely populated areas. We created a master list that included all of the top queries from the United States, Louisiana, and Mississippi and the most strongly associated follow-up terms that derived from the top queries. Directed graphs mapped the initial search term to its top related queries in order of the strength of their association, a number presented in parenthesis in each node (Figure 2, Figure 3, and Figure 4).

**Figure 5:**
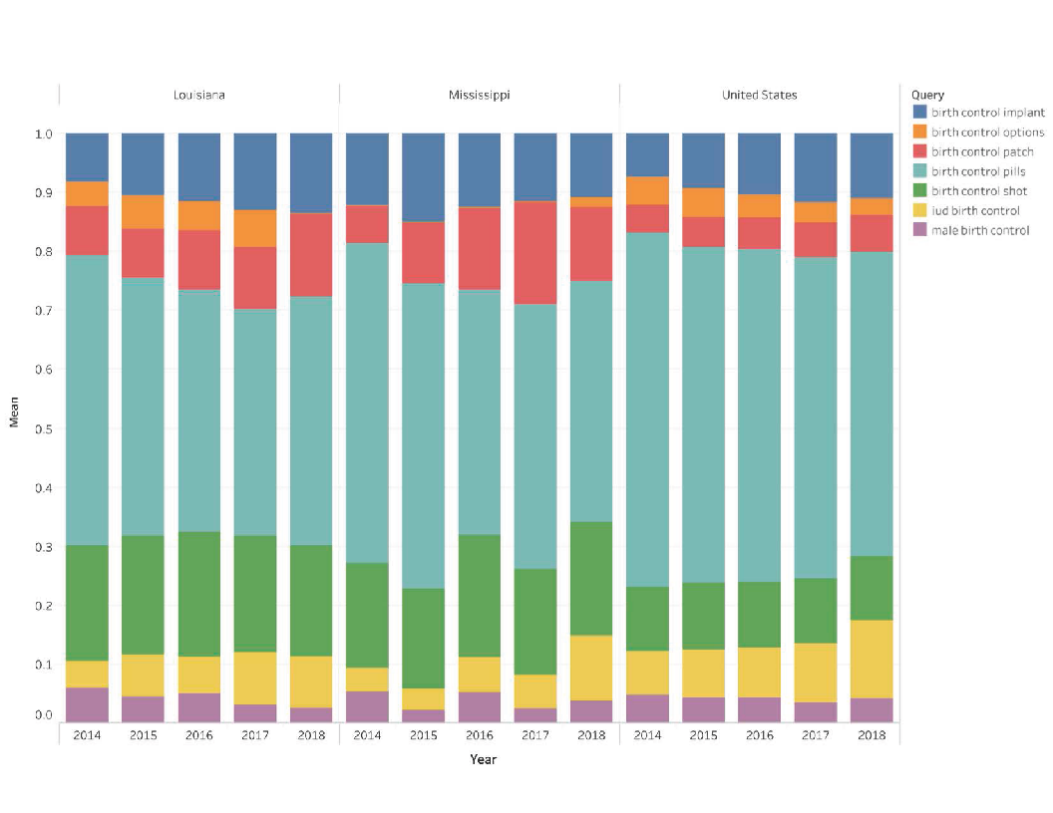
Trends in relative search volumes for top search queries related to birth control in the United States, Louisiana, and Mississippi, 2014-2018; the relative search volume is the proportion of a specific search term among all queries associated with birth control in a given location and year.

To compare the relative search frequencies queries across Louisiana, Mississippi, and the United States, we used the Google Health Trends API. The Google Health Trends API did not give results if there are not enough searches above the privacy threshold matching our parameters. We display the relative search volume of the top queries in each location and year in a bar graph (Figure 5). To establish significant differences in relative search volume searches in years prior to 2017, we estimated 95% confidence intervals of the point estimate of each term. However, these significant differences are reported in the results but not in Figure 5.

**Figure 6:**
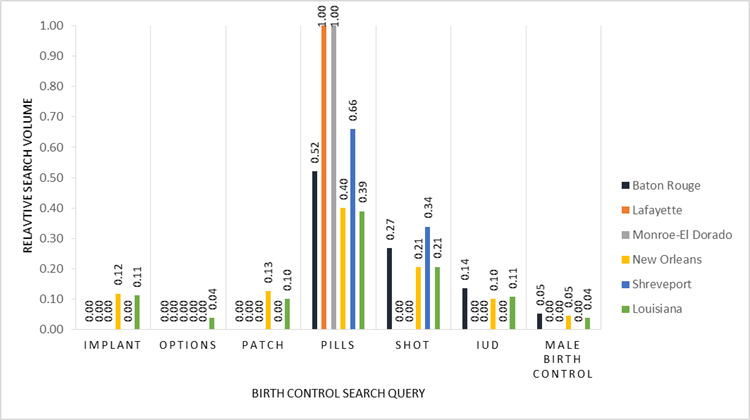
Relative search volume for birth control search queries in Louisiana and in 5 designated marketing areas (DMAs) in Louisiana in 2017. In Louisiana, of the 7 DMAs in total, Alexandria and Lake Charles did not meet the threshold for data extraction and were excluded.

**Figure 7:**
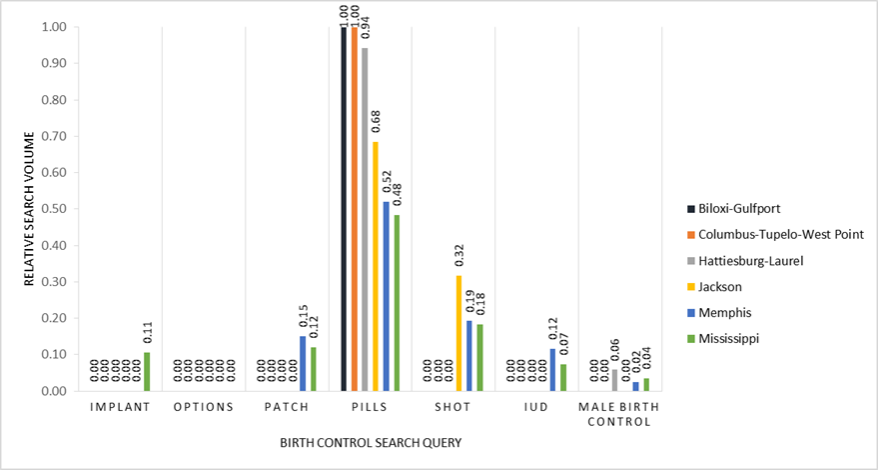
Relative search volume for birth control search queries in Mississippi and in 5 designated marketing areas (DMAs) in Mississippi in 2017. In Mississippi, of the 7 DMAs in total, Greenwood-Greenville and Meridian did not meet the threshold for data extraction and were excluded.

To examine regional differences within each state, we focused on designated market areas (DMAs), which are the smallest geolocations for which Google search data are reported. DMAs, which we acquired from Nielsen Company 2017 data, are the geographic areas in the United States in which local television viewing is measured by Nielsen, an American information, data, and measurement firm [20]. DMA data are essential for any marketer, researcher, or organization seeking to utilize standardized geographic areas [21]. Both Louisiana and Mississippi are divided into 7 DMAs; however, for this study, only 5 DMAs in each state met the Google Health Trends API confidentiality threshold for extracting data. Relative search volume by DMA gave us the rankings of search volume for each birth control method in each DMA as compared to one another when sufficient volume of searches was available in the DMA (Figure 6 and Figure 7). These data allowed us to explore how search interest for each contraceptive method varies between different geographical areas in each of the 2 states.

Some of the data presented in this paper were used to test our methodology for retrieving Google data from APIs as published in Journal of Medical Internet Research (JMIR) research protocols (RR1-10.2196/16543) [18]. This paper presents the final analysis and results, although some of the figures might seem similar to those already published alongside the research protocol. The protocol was also applied to abortion searches on Google in Louisiana and Mississippi in a PLOS One publication by Guendeleman et al [22].

## Results

### Tracking Search Queries on Birth Control

In Figure 2, the directed graph of top queries in the United States as described in the Methods section shows that in 2017, the most popular search query for the term for “birth control” in the United States was birth control pills. The value of 100 for birth control pills denotes that they were most strongly associated with the search term “birth control.” Following birth control pills, other popular search queries were for the birth control shot (injectable contraceptive), the implant, male birth control (referring only to vasectomy), and IUD (intrauterine device; referring to copper IUD).

Figure 3, the directed graph of top queries in Louisiana, shows that Louisiana followed a similar pattern to the United States, except that IUD was not a popular search query associated with birth control in 2017. Meanwhile, the birth control patch was among the top queries associated with birth control in Louisiana, but not found in the United States population as a whole. Figure 4, the directed graph of top queries in Mississippi, shows that in contrast to the wide range of contraceptive methods frequently searched for in the United States and Louisiana, the top searches in Mississippi were circumscribed to birth control pills. In 2018, the top queries for birth control remained the same in Louisiana and Mississippi (data not shown). However, in the United States, while the IUD was among the top queries in 2017, it disappeared from the top query list in 2018 (data not shown). Even though the IUD continued to be searched, it was not part of the top query list associated with birth control.

### Trends in Birth Control Search Traffic Between 2014 and 2018

Unlike search queries on birth control where only those meeting a threshold appeared in the graphs, we employed the Google Health Trends API to include all birth control–related top queries (first row of Figure 2) in the United States to enable comparisons in relative search volume. Relative search volume gives us the proportion of specific search terms among all queries associated with birth control in a given location and year. Figure 5 shows the trends in relative search volume for the birth control top queries during 2014-2018. In the United States, Louisiana, and Mississippi, birth control pills remained the most searched query from 2014 to 2018. While similar relative search volumes through the observed period were seen in the United States and Mississippi, in Louisiana, birth control pills search volume declined from 2014 to 2016 and was similar in 2017 and 2018.

In the United States, Louisiana, and Mississippi, the birth control shot appeared as the second most searched query but not significantly different throughout the period in any of the geolocations (Figure 5). Implants were the third most searched method in Louisiana and Mississippi, while in the United States, it was the IUD. Relative search volume for implants increased over time in Louisiana and the United States, while it remained stable in Mississippi. Increases in relative search volumes for IUDs and the patch were observed in all 3 geolocations in the last 2 years of the studied period (2017-2018).

### State-Level Geographic Differences in Birth Control Search Traffic

**Figure 8:**
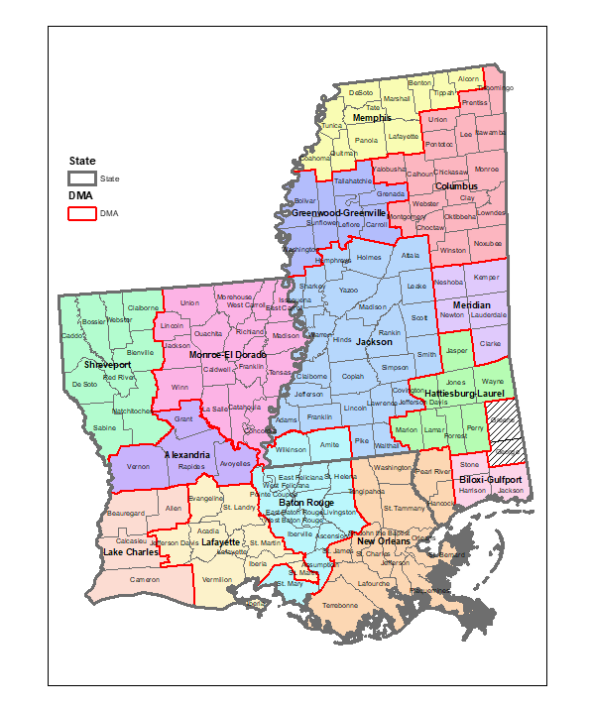
Breakdown of Louisiana and Mississippi counties by designated marketing area (DMA); all 7 DMAs in Mississippi represent aggregated counties or zip codes in the state. In Mississippi, 6 counties (George, Green, Pearl River, Hancock, Amite, and Wilkinson) are located in DMAs that are primarily made up of zip codes in other states and were excluded.

The DMA-level analysis showed variations in search traffic for birth control methods across each state. Figure 8 breaks down Louisiana and Mississippi by county and DMA. In Louisiana, searches for birth control pills were relatively higher in Lafayette and Monroe El Dorado compared to other DMAs, and birth control pills were the only query related to birth control (Figure 6). Regarding searches on birth control shot, the second most common query, Shreveport emerged as the DMA with the relative highest volume followed by Baton Rouge (Figure 6). The New Orleans DMA had the most diverse queries, similar to the broader Louisiana search pattern. In Mississippi, the searches for the pill were much higher in Biloxi-Gulfport and Columbus; Jackson and Memphis DMAs had higher relative search volumes for the birth control shot (Figure 7). Memphis DMA had the most diverse queries, whereas Biloxi-Gulfport and Columbus only had queries on birth control pills. Regarding searches for IUDs, Memphis represented the majority of searches in the state for this method.

## Discussion

### Principal Findings

The internet is growing as a platform to source health information for many users, including birth control, as found in this study. While we do not know why people are searching for birth control information on Google, we surmise that it follows a similar pattern to other online health-seeking behavior, with the search engine providing a starting point for answering basic questions, understanding options available, and seeking health services [1,2,23]. Limited access to information and services through other channels may also force individuals to rely on the internet more frequently. Currently, neither Louisiana nor Mississippi requires coverage of prescription contraception or no-cost contraception coverage [11]. At the same time, while sex education is mandated in Mississippi, the curriculum does not have to be evidence-based or provide information beyond abstinence. In Louisiana, no sex education is mandated, and if provided, it must emphasize abstinence [24]. Lack of access to information and resources has been linked to internet use for information and services. The use of the web may become more important in contraceptive decision making, providing opportunities for individuals to connect with trusted and accurate online resources [9,25]. Experts acknowledge that better-informed choices lead to better reproductive health outcomes [26]. A key factor of preventative health practice is access to information regarding risks to health and promotional measures for improving health status. Meanwhile, the digital age has changed the landscape of health information seeking [27]. A qualitative study to evaluate the process of online health information searching found that participants viewed the internet as a valuable tool for finding health information in order to support their existing health care resources [23]. The authors emphasized knowing what health information people are searching for online can help address knowledge gaps between providers and patients.

We found birth control pills were the most common search query related to birth control across all 3 geolocations. Though we cannot compare searches on birth control to actual use of methods, these findings are supported by a 2017 Guttmacher Institute report assessing state-level estimates of contraceptive use that found that the birth control pill was one of the most commonly used primary methods of contraception among women aged 18-49 years at risk of unintended pregnancy in the United States, ranging from 11% in Alaska to 27% in Massachusetts [28]. In Louisiana and Mississippi, 17.9% and 14.2%, respectively, of women at risk of unintended pregnancy were using birth control pills as their primary method of contraception.

Birth control pills also remained the most popular query throughout the data looking at trends from 2014-2018. Though there have been significant increases in use of long-acting reversible contraception (LARC) in the last decade in the United States [29] and our study showed noticeable increases in searches conducted for LARC over time (2014-2018), actual LARC use in Louisiana and Mississippi is lower than most other states [28]. Louisiana had the lowest use of the IUD at 4.5% of women at risk of unintended pregnancy in the 2017 Guttmacher Institute report. At 7.1% IUD use among women at risk of unintended pregnancy, Mississippi was also on the low end among states; neither Louisiana nor Mississippi had reliable data on implant use. A 2016 study among women of reproductive age in the United States found that 33% of surveyed women were either unaware of LARC (including both IUDs and implants) or had misperceptions about their effectiveness or safety [30]. Another study found there was a sharp increase (21.6%) in the rate of women who chose LARC methods in the 30 days after the 2016 United States presidential election as compared to the 30 days before [31], which corresponded with a spike in social media calling for women to get LARC methods after the election of President Trump given concerns about the future of the Affordable Care Act and contraception coverage [32]. This suggests that Google search patterns may be influenced by mainstream and social media. Additionally, changes to contraceptive care laws and funding, trending news stories, and political events are other factors that might affect online search behaviors.

While we found wide regional variations in search traffic for birth control methods by DMA for both Louisiana and Mississippi, more data collection efforts are needed to identify the factors that are driving differences in search behaviors related to birth control in these localities. However, there were notable differences between DMAs that include larger cities versus the DMAs that were more rural. The New Orleans DMA in Louisiana and the Memphis DMA both contain large cities and also had the most diverse search queries among all DMAs. Though we cannot draw conclusions from these findings, they are important to explore further given both health disparities in rural women [33] and the digital gap between rural and nonrural communities in the United States [34]. Further examination of the regional differences in birth control searches can inform providers and advocates of the birth control interests of the populations in particular geographies. Internet search histories by geography can also be used in combination with service availability and service utilization data to provide a better picture of where the gaps in both services and need for more knowledge exist, including locations to obtain counseling and services.

### Limitations

There are several limitations to account for when interpreting the data and methodology. This study focused on the United States, specifically 2 states — Louisiana and Mississippi. Therefore, applicability outside of the United States is limited. Additionally, there is variability in online access and internet usage across different demographic, socioeconomic, and geographic subpopulations within the United States, such that certain groups of users might be over- or underrepresented among internet search data. We do not know the reasons that prompt individuals to search for contraception information, nor the demographic characteristics of the individuals searching for the topics assessed in this analysis. However, previous research has indicated that female-identifying internet users are more likely to seek health information online [4,35]. We also cannot be certain that searching for a topic online reflects intention to use or current use of contraception or how people perceive or use this information. The Google Trends API used for identifying top queries only shows the queries highly associated with the search term “birth control.” Thus, queries that have weaker associations with birth control are not reported. The Google Health Trends API also does not report relative search volume below a certain threshold (unknown to us). Another limitation is related to interpretation of the DMA data. A DMA region is comprised of counties that form an exclusive geographic area in which the home market television stations hold a dominance of total hours viewed, but these counties are not necessarily located in the same state. For example, the Memphis DMA consists of counties in Mississippi, Arkansas, and Tennessee. In Mississippi, 6 counties (George, Green, Pearl River, Hancock, Amite, and Wilkinson) are located in DMAs that are primarily made up of zip codes in other states and therefore were not included in the analysis. In the Louisiana DMAs, 4 counties were included that are not part of Louisiana. Wilkinson and Amite are located in the Baton Rouge DMA. Pearl River and Hancock are located in the New Orleans DMA. Despite these limitations, the analysis of Google search traffic data presents important insights into the most popular searches related to birth control, information that can be useful for health providers and program implementers in the United States, including those dedicated to providing accurate information about birth control methods.

### Conclusions

The convenience, accessibility, and availability of information on the internet have resulted in increasing numbers of individuals searching for health information online, and as shown in our data from the United States, Louisiana, and Mississippi, this includes searching for information on birth control. Understanding popular Google search queries on birth control can inform in-person discussions initiated by family planning practitioners and inform broader birth control messaging campaigns. Further research is needed to understand the quality and quantity of birth control information on the internet and ensure those searching are getting comprehensive and accurate information on birth control.

## Abbreviations

API: application programming interface
DMA: designated market area
IUD: intrauterine device
JMIR: Journal of Medical Internet Research
LARC: long-acting reversible contraception
